# Analysis of the completeness and consistency of records of violence against indigenous women in the health macro-region of Dourados, Mato Grosso do Sul state, Brazil, 2009-2020

**DOI:** 10.1590/S2237-96222024V33E20231075.EN

**Published:** 2024-05-27

**Authors:** Glênio Alves de Freitas, Gláucia Elisete Barbosa Marcon, James Robert Welch, Cosme Marcelo Furtado Passos da Silva

**Affiliations:** 1 Universidade Federal de Uberlândia, Departamento de Saúde Coletiva, Uberlândia, MG, Brazil; 2 Fundação Oswaldo Cruz, Escritório Regional, Campo Grande, MS, Brazil; 3 Fundação Oswaldo Cruz, Escola Nacional de Saúde Pública Sergio Arouca, Rio de Janeiro, RJ, Brazil

**Keywords:** Violence Against Indigenous Women, Notification, Notifiable Health Conditions Information System, Time Series, Violencia Contra las Mujeres Indígenas, Notificación, Notificación de Sistemas de Información en Salud, Series de Tiempo, Violência Contra a Mulher Indígena, Notificação, Sistema de Informação de Agravos de Notificação, Séries Temporais

## Abstract

**Objective:**

To analyze the temporal trend of completeness and consistency of data on notifications of violence against indigenous women in the health macro-region of Dourados, state of Mato Grosso do Sul, Brazil, between 2009 and 2020.

**Methods:**

An ecological time series study was conducted using data from the Notifiable Health Conditions Information System; Prais-Winsten regression was used to analyze the trend of data completeness and consistency, as well as the proportion of completed and coherent fields.

**Results:**

A total of 2,630 cases were reported; completeness was found to be very poor in the variable “occupation” (48.9%) and poor in the variables “schooling” (68.3%) and “time of occurrence” (67.9%); in the analysis of temporal trends, only the variable “occupation” showed a decreasing trend (p = 0.045).

**Conclusion:**

The data analyzed demonstrated the need for improvement in the completeness of the variables “schooling”, “occupation” and “time of occurrence” of the violent act.

## Study contributions

**Main results:**

There was a progressive increase in notifications over the years. Most of the variables showed regular or excellent completeness and consistency. In the analysis of temporal trend, only the “occupation” variable showed a decreasing trend.

**Implications for services:**

Care for victims of violence is part of the daily routine of health services, and it is essential for health professionals to provide adequate compulsory notification for a comprehensive understanding of the victims’ profile, thus assisting in addressing this issue.

**Perspectives:**

Further studies are needed to understand the factors associated with violence against indigenous women, which could help the development of health promotion actions and violence prevention strategies targeting these women.

## Resumo

**Objetivo:**

Analisar a tendência temporal do grau de completitude e a consistência dos dados de notificação de violência contra as mulheres indígenas da macrorregião de saúde de Dourados, Mato Grosso do Sul, Brasil, entre os anos de 2009 e 2020.

**Métodos:**

Estudo ecológico de série temporal, sobre dados do Sistema de Informação de Agravos de Notificação; utilizou-se a regressão de Prais-Winsten para analisar a tendência da completitude dos dados e sua consistência, a proporção de campos preenchidos e coerentes.

**Resultados:**

Foram notificados 2.630 casos; a completitude revelou-se muito ruim na variável “ocupação” (48,9%) e ruim nas variáveis “escolaridade” (68,3%) e “hora da ocorrência” (67,9%); na análise de tendência temporal, apenas a variável “ocupação” apresentou tendência de redução (p = 0,045).

**Conclusão:**

Os dados analisados demonstraram a necessidade de melhoria na completitude das variáveis “escolaridade”, “ocupação” e “hora da ocorrência” do ato violento.

## Resumen

**Objetivo:**

Analizar la tendencia temporal en el grado de completitud y consistencia de los datos de notificación sobre violencia contra mujeres indígenas en la macrorregión sanitaria de Dourados, Mato Grosso do Sul, Brasil, entre los años 2009-2020.

**Métodos:**

Estudio de series de tiempo ecológicas con datos del Sistema de Información de Enfermedades de Declaración Obligatoria; se utilizó la regresión de Prais-Winsten para analizar la tendencia de completitud; para mantener la coherencia, se utilizó la proporción de campos completados y coherentes.

**Resultados:**

Se notificaron 2.630 casos; la exhaustividad fue muy pobre en la variable ocupación (48,9%) y pobre en las variables educación (68,3%) y tiempo de ocurrencia (67,9%); en el análisis de tendencia temporal, sólo la variable ocupación mostró una tendencia decreciente (p = 0,045).

**Conclusión:**

Los datos analizados demuestran la necesidad de mejorar la completitud de las variables educación, ocupación y tiempo de ocurrencia del acto violento.

## INTRODUCTION

Violence is a historical and social phenomenon present in humankind. Considered a health problem, it demands specific public policies, organization of practices and services, given its power to affect individual and collective health.^1^ According to the World Health Organization’s (WHO) World Report on Violence and Health, violence can be prevented and its impacts reduced through strategies such as promoting gender equality, restricting the availability and reduction of the harmful use of alcohol, as well as access to firearms, in addition to changing cultural and social norms that favor them.^2^ In order to reduce and prevent cases of violence, it is necessary to implement a victim assistance network, with a multidisciplinary approach that intervenes in cases and prevents recurrences.^2^

According to the WHO, one in three women worldwide has experienced some form of physical or sexual violence by an intimate partner or sexual violence by a non-partner.^3^ In Brazil, according to occurrence bulletins and femicide qualification by civil police stations, in the first half of 2022, nearly 700 women were victims of this fatal form of violence, constituting four murders every day, on average. Compared to data from the first half of 2019, released by the Brazilian Forum of Public Security, the increase in femicide in the country was 10.8% in 2022.^4^

In the context of the indigenous population, indigenous women are vulnerable to some form of violence due to increased contact with the urban environment, which has changed their traditional social and economic structure and contributed to the adoption of new habits and, consequently, tumultuous gender relations.^5^ A descriptive report, based on data from Public Safety Canada, assessing 516 homicides that occurred in 2014, showed that indigenous women in that country were 6.5 times more likely to be killed than non-indigenous women.^6^

In the state of Mato Grosso do Sul, located in the Midwest region of Brazil, high population density in some indigenous lands, conflicts over land ownership, contact with the non-indigenous population, the degradation of the original landscape, as well as the introduction of illicit drugs and alcohol, have contributed to the occurrence of violence against Guarani and Kaiowá women.^5^

In addressing violence against women, it is the responsibility of the health services to provide care and compulsory notification of these cases by health professionals, in a specific form designed for recording this violence. Once filled out and entered into the Notifiable Health Condition Information System (*Sistema de Informação de Agravos de Notificação* - SINAN), this form generates data for planning public policies aimed at preventing and reducing these occurrences. However, the invisibility of violence against women in health services means that many cases go unreported, or when they are, the reports have several unfilled fields.^7^

There is a scarcity of epidemiological studies that evaluate the SINAN database on violence against indigenous women. Thus, the completeness of a variable, characterized by the degree to which it is filled with a non-null value, and its consistency, defined by its coherence in relation to another variable, contributes to better surveillance of this health condition.^8^ ^),(^ ^9^

The study aimed to analyze the temporal trend of completeness and consistency of data on notifications of violence against indigenous women in the health macro-region of Dourados, state of Mato Grosso do Sul, Brazil, between 2009 and 2020.

## METHODS

This was an ecological time series study that assessed the temporal trend of the degree of completeness and consistency of cases of violence against indigenous women aged 10 years and older, living in the health macro-region of Dourados, state of Mato Grosso do Sul. The data evaluated were reported on SINAN from 2009 to 2020.

The health macro-region of Dourados is located in the southern region of the state of Mato Grosso do Sul and borders Paraguay. In 2022, this macro-region comprised a population of nearly 1 million inhabitants, spread across 33 municipalities,^10^ and approximately 60 thousand people living in indigenous lands, predominantly from the Guarani and Kaiowá ethnic groups, according to the report of the Special Indigenous Health District of Mato Grosso do Sul (*Distrito Sanitário Especial Indígena do Mato Grosso do Sul* - DSEI/MS) for 2023. Primary Health Care (PHC) for this population is provided by the DSEI/MS through health centers in their respective territories.^11^

The unidentified records from SINAN were provided by the state Center for Strategic Information in Health Surveillance (*Centro de Informações Estratégicas de Vigilância em Saúde* - CIEVS) in September 2022. SINAN is a national health information system, decentralized to municipalities, where data on compulsorily notifiable health conditions are entered. Notifications of violence are carried out by healthcare professionals and forwarded to municipal epidemiological surveillance services, which are responsible for entering the data into the system.

The analysis of the SINAN database on violence against indigenous women was evaluated for its completeness and consistency. Completeness is characterized by the degree of completion of the variable under analysis, measured by the proportion of notifications with categories other than those indicating the absence of data:^8^ fields of variables classified as “unknown” or blank were considered unfilled. Consistency, on the other hand, can be defined as the proportion in which the relationship between two selected variables presents coherent values. An example of inconsistent data is the notification of violence against an eight-year-old child with complete high school reported in the notification.

The completeness analysis took into consideration 50 variables from the violence notification/investigation form, namely:

a) Data on the individual assisted
1. Schooling
2. Marital status
3. Occupation
4. Pregnancy status
5. Disability
b) Incident data
6. Place of occurrence
7. Time of occurrence
8. Previous occurrences
9. Physical violence
10. Sexual violence
11. Psychological violence
12. Torture
13. Neglect/abandonment
14. Human trafficking
15. Use of body force/beating
16. Hanging
17. Use of blunt object
18. Use of sharp object
19. Use of hot substance/object
20. Poisoning/intoxication
21. Use of firearms
22. Threat
c) Data on the perpetrator
23. Number of individuals involved
24. Father
25. Mother
26. Stepfather
27. Spouse
28. Former spouse
29. Boyfriend
30. Ex-boyfriend
31. Acquaintance
32. Stranger
33. Caregiver
34. Employer/boss
35. Institutional relationship person
36. Sex of the perpetrator
37. Suspected alcohol use
d) In cases of sexual violence
38. Sexually transmitted diseases (STDs) prophylaxis
39. Human immunodeficiency virus (HIV) prophylaxis
40. Hepatitis B prophylaxis
41. Blood collection
42. Semen collection
43. Vaginal secretion collection
44. Emergency contraception
45. Lawful abortion
e) Case progression and referral
46. Women’s Police Station
47. Other police stations
48. Public Prosecutor’s Office
49. Social Assistance Reference Center (Centro de Referência de Assistência Social - CRAS)
50. Institute of Forensic Medicine (*Instituto Médico Legal* - IML)

The selection of these variables was based on the following factors, according to the SINAN notification form: they are mandatory for data entry; they are considered essential for epidemiological and operational analysis; they are considered important for the case surveillance process; and they contribute to the construction of epidemiological indicators.

Regarding consistency, the following relationships between variables were analyzed:

a) reproductive age (10 to 49 years) versus pregnancy status (not applicable);

b) disability/disorder (yes) versus type of disability/disorder (no);

c) psychological violence (yes) versus type of aggression (excluding threats);

d) sexual violence (yes) versus type of sexual violence (unknown); and

e) sex of the perpetrator of the aggression (male) versus relationship (mother).

Consistency was analyzed only for the completed variables. The percentage was obtained by dividing the number of consistent relationships divided by the total number of relationships between the considered variables.

The notification years were grouped into biennia: 2009-2010; 2011-2012; 2013-2014; 2015-2016; 2017-2018; 2019-2020. In the analysis of completeness and consistency, the percentage of correctly filled variables (for each period) and the total percentage were calculated. For completeness, the following categories were considered: excellent, greater than or equal to 95.0%; good, from 90.0% to 94.9%; regular, from 70.0% to 89.9%; poor, from 50.0% to 69.9%; and very poor, less than or equal to 49.9%.^8^ For consistency, the considered categories were: excellent, greater than or equal to 90.0%; regular, from 70.0% to 89.9%; and poor, less than 70.0%.^12^

The annual percentage change (APC) was calculated using Prais-Winsten linear regression, with the biennium as the independent variable (y) and the percentage of completeness as the dependent variable (x).^13^ A significance level of 5% was used in statistical tests and a 95% confidence interval (95%CI) was calculated. A decreasing trend was considered when the 95%CI showed negative values, an increasing trend was considered when the 95%CI showed positive values, and stability when the 95%CI showed both positive and negative values.^14^ The analyses were performed using the R statistical software, version 4.1.2.

The study project was approved by the Research Ethics Committee of the Escola Nacional de Saúde Pública Sergio Arouca/Fundação Oswaldo Cruz (CEP/ENSP/Fiocruz), Opinion No. 5,274,177; and by the National Research Ethics Committee/National Health Council (*Comissão Nacional de Ética em Pesquisa/Conselho Nacional de Saúde* - CONEP/CNS), Opinion No. 5,469,695. Mato Grosso do Sul State Health Department (SES/MS) signed the consent letter for this research to be conducted.

## RESULTS

**Figure 1:**
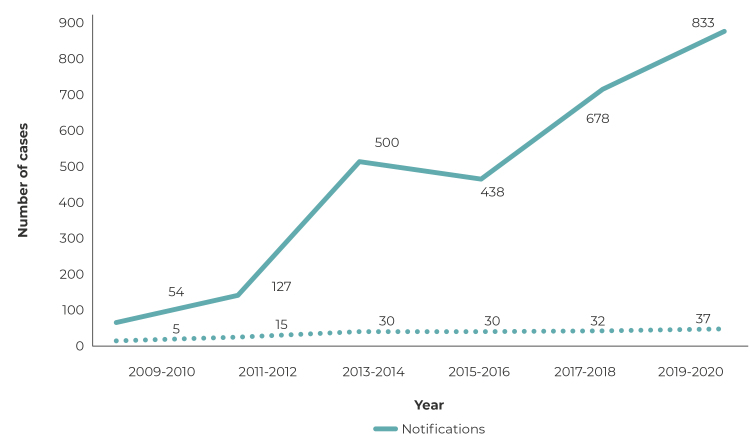
Distribution of the number of notifications of violence against indigenous women in the Notifiable Health Conditions Information System (SINAN) and institutions that reported violence, health macro-region OF Dourados, Mato Grosso do Sul state, Brazil 2009-2020

Source: SINAN.

Between 2009 and 2020, a total of 2,630 notifications of violence against indigenous women were recorded in the health macro-region of Dourados, state of Mato Grosso do Sul, all of which were analyzed in this study. There was an increase in the number of notifications in the period from 2009 to 2020, ranging from 54 in the first biennium (2009-2010) to 833 in the last biennium (2019-2020); as well as an increase in the number of notifying institutions, from 5 in the first biennium to 37 in the last biennium (Figure 1). Women aged 10 to 19 years accounted for the majority of registrations for the entire period (33.0%), as shown in Table 1.

**Table 1:** Number and percentage of notifications of violence against indigenous women aged 10 years and older, according to biennium and age group, health macro-region of Dourados, Mato Grosso do Sul state, Brazil, 2009-2020

Age (years)	2009-2010 (N = 54)	2011-2012 (N = 127)	2013-2014 (N = 500)	2015-2016 (N = 438)	2017-2018 (N = 678)	2019-2020 (N = 833)	Total (N = 2.630)
	N (%)	N (%)	N (%)	N (%)	N (%)	N (%)	N (%)
10-19	15 (27.8)	36 (28.3)	171(34.2)	149 (34.0)	257 (37.9)	241 (28.9)	869 (33.0)
20-29	15 (27.8)	35 (27.6)	108 (21.6)	104 (23.7)	180 (26.5)	265 (31.8)	707 (26.9)
30-39	7 (13.0)	30 (26.6)	103 (20.6)	100 (22.8)	130 (19.2)	156 (18.7)	526 (20.0)
40-49	4 (7.4)	15 (11.8)	58 (11.6)	39 (8.9)	56 (8.3)	94 (11.3)	266 (10.1)
≥ 50	13 (24.0)	11 (8.7)	60 (12.0)	46 (10.6)	55 (8.1)	77 (9.3)	262 (10.0)

Source: Notifiable Health Conditions Information System (*Sistema de Informação de Agravos de Notificação* - SINAN).

The lowest completeness was recorded for the variable “occupation” (48.9%), classified as very poor. The variables “schooling” (68.3%) and “time of occurrence” (67.6%) showed poor completeness. The highest degrees of completeness, classified as good, were observed for the variables “physical violence” (93.5%), “number of individuals involved” (93.6%), “sex of the perpetrator” (90.5%), “prophylaxis for STDs” (90.0%) and “blood collection” (90.0%). Completeness was considered regular for 42 variables. As for the analysis of temporal trend, only the variable “occupation” showed a decreasing trend in the period 2009-2020 (p = 0.045); for 31 variables, the temporal trend was increasing; and for 18 variables, the trend was stable (Table 2).

**Table 2:** Number, percentage and temporal trend of completeness of fields in the notification/investigation form for violence against indigenous women aged 10 years and older, health macro-region Dourados, Mato Grosso do Sul state, Brazil, 2009-2020

Variables	2009-2010 (N = 54)	2011-2012 (N = 127)	2013-2014 (N = 500)	2015-2016 (N = 438)	2017-2018 (N = 678)	2019-2020 (N = 833)	Completeness (%)	Degree of completeness	APC^a^ (_95%_CI^b^)	p-value^c^	Trend
	N %	N %	N %	N %	N %	N %					
Data on the individual assisted											
Schooling	35 63.0	73 57.5	306 61.2	256 58.4	507 74.8	621 74.5	68.3	Poor	3.18 (-0.32;6.69)	0.065	Stability
Marital status	42 77.8	109 85.8	448 89.6	339 77.4	529 78.0	703 84.4	82.5	Regular	0.29 (-3.77;3.18)	0.826	Stability
Occupation	37 68.5	104 81.9	416 83.2	136 31.1	257 37.9	335 40.2	48.9	Very poor	-10.23 (-20.11;-0.35)	0.045	Decreasing
Pregnancy status	45 83.3	94 74.0	414 82.8	369 84.2	606 89.4	783 94.0	87.9	Regular	3.20 (1.24;5.17)	0.010	Increasing
Disability	44 81.5	108 85.0	438 87.6	334 76.3	540 79.6	742 89.1	83.9	Regular	0.00 (-3.12;3.12)	0.998	Stability
Incident data											
Place of occurence	43 79.6	108 85.0	433 86.6	356 81.3	588 86.7	739 88.7	86.2	Regular	1.13 (-0.38;2.65)	0.106	Stability
Time of occurrence	35 64.8	67 52.8	328 65.6	263 60.0	488 72.0	596 71.5	67.6	Poor	3.05 (1.18;4.92)	0.016	Increasing
Previous occurrence	40 74.1	81 63.8	355 71.0	254 58.0	519 76.5	640 76.8	71.8	Regular	1.30 (-2.72;5.43)	0.418	Stability
Physical violence	51 94.4	122 96.1	487 97.4	402 91.8	625 92.2	771 92.6	93.5	Good	0,83 (-1.99;0.32)	0.115	Stability
Sexual violence	46 85.2	92 72.4	433 86.6	370 84.5	606 89.4	771 92.6	88.1	Regular	2.99 (1.35;4.64)	0.007	Increasing
Psychological violence	44 81.5	93 73.2	427 85.4	371 84.7	596 87.9	769 92.3	87.5	Regular	3.13 (1.93;4.34)	0.001	Increasing
Torture	43 79.6	86 67.7	418 83.6	366 83.6	593 87.5	769 92.3	86.5	Regular	4.04 (2.29;5.79)	0.003	Increasing
Neglect/abandonment	43 79.6	86 67.7	422 84.4	368 84.0	593 87.5	769 92.3	86.7	Regular	4.03 (2.27;5.08)	0.003	Increasing
Human trafficking	43 79.6	85 66.9	424 84.8	368 84.0	594 87.6	771 92.6	86.9	Regular	4.17 (2.03;6.04)	0.003	Increasing
Use of body force/beating	48 88.9	108 85.0	443 88.6	388 88.6	609 89.8	760 91.2	89.6	Regular	0.91 (0.28;1.54)	0.015	Increasing
Hanging	42 77.8	87 68.5	414 82.8	360 82.2	589 86.9	762 91.5	85.7	Regular	3.96 (2.60;5.31)	0.001	Increasing
Use of blunt object	42 77.8	88 69.3	417 83.4	364 83.1	594 87.6	762 91.5	86.2	Regular	3.93 (2.66;5.25)	0.001	Increasing
Use of sharp object	43 79.6	91 71.7	435 87.0	369 84.2	598 88.2	761 91.4	87.3	Regular	3.41 (2.08;4.74)	0.002	Increasing
Use of hot substance/object	42 77.8	88 69.3	411 82.2	363 82.9	589 86.9	762 91.5	85.7	Regular	3.89 (2.54;5.24)	0.001	Increasing
Poisoning/intoxication	42 77.8	88 69.3	408 81.6	362 82.6	588 86.7	762 91.5	85.6	Regular	3.87 (2.49;5.25)	0.001	Increasing
Use of firearms	42 77.8	87 68.5	409 81.8	360 82.2	588 86.7	761 91.4	85.4	Regular	3.95 (2.51;5.38)	0.001	Increasing
Threat	45 83.3	86 67.7	406 81.2	355 81.1	590 87.0	760 91.2	85.2	Regular	3.35 (0.82;5.88)	0.021	Increasing
Data on the perpetrator											
Number of individuals involved	50 86.2	121 93.8	493 94.1	387 92.1	642 94.8	642 94.8	93.6	Good	1.12 (-0.26;2.52)	0.088	Stability
Father	46 79.3	94 72.9	436 3.2	354 84.3	604 89.2	604 89.2	84.5	Regular	3.33 (2.22;4.44)	0.001	Increasing
Mother	46 79.3	92 71.3	440 4.0	356 84.8	604 89.2	604 89.2	85.0	Regular	3.53 (2.12;4.93)	0.002	Increasing
Stepfather	46 79.3	93 72.1	431 82.3	354 84.3	604 89.2	604 89.2	84.5	Regular	3.44 (2.10;4.77)	0.002	Increasing
Spouse	46 79.3	102 79.1	457 87.2	364 86.7	619 91.4	619 91.4	87.8	Regular	3.00 (2.36;3.63)	< 0.001	Increasing
Former-spouse	46 79.3	93 72.1	433 2.6	348 2.9	606 89.5	606 89.5	84.4	Regular	3.47 (2.34;4.60)	0.001	Increasing
Boyfriend	45 77.6	92 71.3	429 1.9	348 2.9	604 89.2	604 89.2	83.9	Regular	3.73 (2.66;4.81)	< 0.001	Increasing
Ex-boyfriend	45 77.6	94 72.9	429 1.9	348 2.9	604 89.2	604 89.2	84.0	Regular	3.52 (2.67;4.38)	< 0.001	Increasing
Acquaintance	45 77.6	94 72.9	443 4.5	349 83.1	610 90.1	610 90.1	85.2	Regular	3.69 (3.05;4.34)	< 0.001	Increasing
Stranger	45 77.6	94 72.9	437 3.4	362 86.2	606 89.5	606 89.5	85.4	Regular	3.62 (2.24;4.99)	0.001	Increasing
Caregiver	45 77.6	91 70.5	434 2.8	351 83.6	602 88.9	602 88.9	84.2	Regular	3.75 (2.53;5.00)	0.001	Increasing
Employer/boss	45 77.6	92 71.3	433 2.6	351 83.6	602 88.9	602 88.9	84.2	Regular	3.67 (2.54;4.79)	< 0.001	Increasing
Institutional relationship person	45 77.6	92 71.3	433 82.6	352 83.8	607 89.1	607 89.1	84.5	Regular	3.67 (2.54;4.79)	< 0.001	Increasing
Sex	51 94.4	112 88.2	466 93.2	382 87.2	620 91.4	754 90.5	90.5	Good	-0.25 (-1.02;0.51)	0.405	Stability
Suspected alcohol use	40 69.0	92 71.3	426 81.3	328 78.1	582 86.0	582 86.0	81.2	Regular	3.72 (2.81;4.63)	< 0.001	Increasing
In cases of sexual violence											
STD^d^ prophylaxis	50 92.6	111 87.4	424 84.8	398 90.9	616 90.9	768 92.2	90.0	Good	0.43 (-1.70;2.58)	0.600	Stability
HIV^e^ prophylaxis	49 90.7	110 86.6	422 84.4	398 90.9	616 90.9	768 92.2	89.8	Regular	0.81 (-1.09;2.71)	0.302	Stability
Hepatitis B prophylaxis	49 90.7	111 87.4	422 84.4	399 91.1	616 90.9	768 92.2	89.9	Regular	0.75 (-1.08;2.58)	0.318	Stability
Blood collection	49 90.7	112 88.2	422 84.4	400 91.3	616 90.9	768 92.2	90.0	Good	0.69 (-1.07;2.46)	0.336	Stability
Semen collection	49 90.7	110 86.6	423 84.6	396 90.4	616 90.9	767 92.1	89.8	Regular	0.75 (-1.13;2.63)	0.330	Stability
Vaginal secretion collection	40 74.1	108 85.0	416 83.2	378 86.3	586 86.4	732 87.9	85.9	Regular	1.94 (0.47;3.42)	0.021	Increasing
Emergency contraception	40 74.1	108 85.0	416 83.2	377 86.1	586 86.4	735 88.2	86.0	Regular	1.97 (0.53;3.41)	0.018	Increasing
Lawful abortion	40 74.1	108 85.0	416 83.2	374 85.4	585 86.3	732 87.9	85.7	Regular	1.90 (0.45;3.34)	0.021	Increasing
Case progression and referral											
Women’s police station	41 75.9	66 52.0	345 69.0	301 68.7	549 81.0	768 92.2	78.0	Regular	5.37 (-0.01;10.77)	0.051	Stability
Other police stations	43 79.6	70 55.1	348 69.6	300 68.5	548 80.8	768 92.2	79.0	Regular	4.40 (-1.51;10.39)	0.107	Stability
Public Prosecutor’s office	42 77.8	64 50.4	334 66.8	302 68.9	545 80.4	768 92.2	78.1	Regular	5.28 (-1.03;11.60)	0.080	Stability
CRAS^f^	42 77.8	61 48.0	334 66.8	303 69.2	547 80.7	768 92.2	78.0	Regular	5.66 (-0.72;12.06)	0.069	Stability
IML^g^	42 77.8	63 49.6	338 67.6	303 69.2	551 81.3	768 92.2	78.0	Regular	5.51 (-0.66;11.68)	0.068	Stability

APC: Annual percentage change; b) 95%CI: 95% confidence interval; c) P-value: P-value, estimated by means of the Prais-Winsten regression; d) STD: Sexually transmitted disease; e) HIV: Human immunodeficiency virus; f) CRAS (Centro de Referência de Assistência Social): Social Assistance Reference Center; g) IML (Instituto Médico Legal). Forensic Medical Institute.

Regarding consistency, the relationships between (i) reproductive age (10 to 49 years) and pregnancy status (not applicable), (ii) psychological violence (yes) and the types of aggression (excluding threats) and (iii) the sex of the perpetrator (male) and the relationship (mother) showed regular consistency. Excellent consistency was observed in the relationship between (i) disability/disorder (no) and type of disability/disorder (yes), and between (ii) sexual violence (yes) and type of sexual violence (unknown) (Table 3).

**Table 3:** Number and percentage of consistency of variables selected in the notification/investigation form for violence against indigenous women aged 10 years and older, according to biennium, health macro-region of Dourados, Mato Grosso do Sul state, Brazil, 2009-2020

Variables	2009-2010	2011-2012	2013-2014	2015-2016	2017-2018	2019-2020	Total percentage of consistency	Classification
	N (%)	N (%)	N (%)	N (%)	N (%)	N (%)	N (%)	
Reproductive age (10-49 years old) *versus* Pregnancy status (not applicable);	25/38 (65.8)	85/96 (88.5)	350/393 (89.1)	285/34 (82.6)	514/585 (87.9)	630/727 (86.7)	1,889 (86.5)	Regular
Disability/disorder (no) *versus* Type of disability/disorder (yes)	53 (100.0)	123 (100.0)	490 (100.0)	431 (100.0)	667 (100.0)	812 (100.0)	2,576 (100.0)	Excellent
Psychological violence (yes) *versus* Type of aggression (excluding threats)		1 (100.0)	2 (100.0)	1/3 (33.33)	7/9 (77.8)	9/12 (75.0)	20 (74.1)	Regular
Sexual violence (yes) *versus* Type of sexual violence (unknown)	8/8 (100.0)	20/23 (87.0)	93/94 (98.9)	73/81 (90.1)	94/105 (89.5)	92/96 (95.8)	380 (93.4)	Excellent
Sex of the perpetrator of the aggression (male) *versus* Relationship (mother)		1 (100.0)	2 (100.0)	4 (100.0)	8 (88.9)	1 (50.0)	16 (88.6)	Regular

## DISCUSSION

The results of this study showed an increase in notifications over the 12 years selected, from 2009 to 2020. The percentage of completeness was classified as regular for the majority of the variables considered. In the analysis of the temporal trend of completeness according to biennia, only the variable “occupation” showed a decreasing trend. Consistencies remained from regular to excellent quality. During the period, there was an increasing trend in the completeness for 31 variables.

The increase in the number of notifications may be related to the fact that, as of 2010, violence was included in the compulsory notification list of diseases in sentinel health units, in accordance with the Ministry of Health Ordinance MS/GM No. 2,472, dated August 31, 2010; and from the following year, 2011, with the publication of Ordinance MS/GM No. 104, dated January 25, 2011, violence became part of the list of National Compulsory Notification List of Diseases.^15^ ^),(^ ^16^ With the creation of the Special Secretariat for Indigenous Health (*Secretaria Especial de Saúde Indígena* - SESAI), the organization of the Indigenous Special Health Districts (*Distritos Sanitários Especiais Indígenas* - DSEIs) and the inclusion of multi-professional healthcare teams, two factors would have contributed to the increase in notifications: (i) increased efforts for violence notifications within DSEIs, since 2013, and (ii) the publication of Ordinance No. 1,271, dated June 6, 2014, which was responsible for the implementation, in the same year, of the SINAN notification units in the indigenous territory.^17^ ^),(^ ^18^

The increase in the number of notifications may be related to the increase in the number of notifying units registered with the National Health Establishment Registry (*Cadastro Nacional de Estabelecimentos de Saúde* - CNES). A study conducted in the state of Rio de Janeiro, between 2009 and 2016, aimed at analyzing the consistency of 103,841 notifications of interpersonal and self-inflicted violence, found a 284% increase in notifications over the period, an increase associated with better structuring of notifying units.^19^ In Belém, capital city of the state of Pará, there was also an increase in notifications of violence alongside the increase in the number of notifying units between 2009 and 2011.^20^ Therefore, the increase in the number of notifying units, associated with a better structure, staffed with trained professionals who are sensitized to the issue of violence, may contribute to the increase in notifications.

Taking into consideration the target population group of this study, the highest number of notifications occurred among women aged 10 to 19 years. In the general population, one of the factors that can contribute to the increase in notifications of violence against this age group is the awareness of professionals for their reporting; among indigenous women, specifically within the context of the ethnic groups studied, the first menstrual period (menarche) marks a rite of passage where the child becomes a woman, allowing for intimate-sexual relationship and childbearing.^21^ As a result of this new status, indigenous women aged 10 to 15 years are subject to potential violence perpetrated by intimate partners: boyfriends, spouses, ex-boyfriends, ex-spouses. In this present study, 409 indigenous pregnant women were reported as victims of violence in this age group, with physical violence being the most frequent, with 226 cases, followed by sexual violence with 141 cases. Of the total number of pregnant women, 258 indigenous women (63.1%) were between 11 and 15 years of age (data not shown).

With regard to the occurrence data block, the variable “physical violence” showed excellent completeness, the time of the occurrence showed poor completeness, and the other variables showed regular completeness. Collecting information on notifications of violence, such as the place and time of occurrence, type of violence and whether there was recurrence, is important for mapping cases and providing security and protection actions for victims.^22^ According to a study conducted in the city of Amambaí, also in the state of Mato Grosso do Sul, between 2007 and 2013, among the Guarani and Kaiowá ethnic groups, it was common for reports to be made to institutions by close relatives or village leaders, not by the victim himself.^5^ However, this trend of reports being based on secondary sources may contribute to the reduced completeness of information about some variables. On the other hand, the high completeness of the variable “physical violence” may be associated with the fact that it is easily recognizable, due to its visible marks on the victims.

Health services are essential for monitoring violence and should be part of the comprehensive care and protection network for people experiencing violence, involving Social Assistance, Education, Public Security, the Judiciary, the Guardianship Council and civil society. Among the indigenous population, for the referral of victims to social protection agencies in order to resolve cases of violence, in addition to the aforementioned agencies, it is important to involve the sector responsible for indigenous policy, social control, and indigenous associations and collectives.^23^

Moreover, concerning indigenous people, the low completeness identified in the evolution and referral block of cases may be related to professionals’ unpreparedness to handle violence cases in this population, either due to language barrier or due to the lack of professional training for intercultural encounters; or even for the difficulty indigenous women face in accessing other services in the network. such as the Judiciary or Public Security, given the challenging access to some indigenous lands and reserves. According to the Violence Mapping, conducted by the Guarani and Kaiowá indigenous women’s collective in the southern region of Mato Grosso do Sul, many indigenous women experience domestic violence but not all of them receive support and/or shelter.^24^ Women do not report such violence because they have nowhere to go, do not know how to start over or have no prospects other than their ethnic-social situation limited horizon. Those who receive the necessary support and shelter still depend on the transportation provided by SESAI, the National Foundation for Indigenous Peoples (*Fundação Nacional dos Povos Indígenas* - FUNAI) and the Guardianship Council, among others.^24^

Referring victims of violence to other services in the protection network is part of the process of comprehensive care, contributing to the humanization of care.^25^ When providing care, it is the responsibility of healthcare professionals to detect possible violence, provide support, care, notification and referral of the victim to other services in the protection network.^25^ The incompleteness of these data makes it difficult to analyze the care flow in the protection network: its absence can either be the result of not completing the specific field - even if the woman has been referred to the protection network - or can be related to the victim not being referred to other services.

Currently, indigenous peoples have limited access to comprehensive and specialized health care. Most of the institutions are located in cities, far from indigenous lands and reserves, making referral and continuity of care difficult.^26^ ^),(^ ^27^ In addition to the distance from the services of the women’s protection network, this population faces challenges in complying with the legal requirements related to violence: police officers, when requested, are not always able to be present in the indigenous territory; and in certain situations, it is women who cannot communicate with the police force because the territory lacks telephone signal.^24^

The consistency analysis demonstrates the coherence of information in the notifications of violence against indigenous women, allowing for data collection through the association between selected variables. A similar result was observed in a study conducted in Recife, the capital city of the state of Pernambuco, between 2009 and 2012, evaluating the completeness, consistency and duplicity of violence notification records on SINAN.^28^

A factor that can compromise the notification of violence against women is the perception among professionals that domestic violence is exclusively related to Public Security and the Judiciary, with no need for involvement from Public Health.^29^ Another relevant point to take into consideration is the feedback to the notifiers by the epidemiological surveillance team. Feedback on the profile of reported cases, data quality and its implications can facilitate the improvement of information quality, as professionals responsible for reporting violence, upon realizing its effectiveness and results, may feel more motivated.^28^ Furthermore, in the care of indigenous peoples, sociocultural differences between healthcare professionals and indigenous victims of violence may hinder the process of reporting cases.

In order to improve the quality of completion of the notification form, it is important to provide continuing education for the responsible professionals, sensitizing them to the importance of generating quality information.^30^ The lack of notification channels in places where services are provided and where it is consolidated negatively influences data quality. In addition, many data are obtained from medical records, which sometimes may not contain all the necessary information.^30^

A limitation of this study is the use of unidentified SINAN data in the assessment of completeness and consistency, which makes it impossible to observe duplicate notifications. Another limitation to highlight is the absence of stratification of indigenous peoples by ethnicity in the violence notification/investigation form, which makes it impossible to analyze this variable in identifying the ethnic group most vulnerable to violence, which would contribute to targeting specific actions according to each group.

This study was crucial in identifying the need to improve the quality of notifications of violence against indigenous women in Dourados, Mato Grosso do Sul. However, the ethnic and cultural diversity of the Brazilian indigenous population, as well as the regionalization of health service management, prevent the extrapolation of the findings to other Brazilian regions. Nevertheless, the study serves as a guidance for further investigations into the quality of reported data in other indigenous territories.

It can be concluded that the data evaluated in this study are adequate for making inferences and epidemiological analysis of cases of violence in the region, although improvements in the completeness of the variables “schooling”, “occupation” and “time of occurrence” are needed. In order to obtain consistent and high-quality data, it is necessary for the Ministry of Health and the SESAI, along with municipal and state health departments, to adopt measures aimed at improving the completion of violence notification/investigation form, such as continuing education for professionals, training on how to deal with intercultural situations, raising awareness about the importance of correctly filling out the forms, epidemiological surveillance actions through case investigation, and periodic analysis of databases to identify inconsistencies and incompleteness. Generating quality data can contribute to assessing the magnitude and determining violence against indigenous women in the health macro-region of Dourados, Mato Grosso do Sul, as well as assisting in the development of public policies addressing this issue.
